# Research Models to Mimic Necrotizing Enterocolitis and Inflammatory Bowel Diseases: Focus on Extracellular Vesicles Action

**DOI:** 10.1093/stmcls/sxad068

**Published:** 2023-09-09

**Authors:** Miriam Duci, Ludovica De Cesare, Agner Henrique Dorigo Hochuli, Maurizio Muraca, Mara Cananzi, Piergiorgio Gamba, Francesco Fascetti-Leon, Michela Pozzobon

**Affiliations:** Department of Women’s and Children’s Health, University of Padova, Padova (PD) - Veneto, Italy; Stem Cells and Regenerative Medicine Lab, Foundation Institute of Pediatric Research Città della Speranza, Padova (PD) - Veneto, Italy; Pediatric Surgery Unit, Department of Women’s and Children’s Health, Padova University Hospital, Padova (PD) - Veneto, Italy; Stem Cells and Regenerative Medicine Lab, Foundation Institute of Pediatric Research Città della Speranza, Padova (PD) - Veneto, Italy; Department of Women’s and Children’s Health, University of Padova, Padova (PD) - Veneto, Italy; Stem Cells and Regenerative Medicine Lab, Foundation Institute of Pediatric Research Città della Speranza, Padova (PD) - Veneto, Italy; Department of Women’s and Children’s Health, University of Padova, Padova (PD) - Veneto, Italy; Stem Cells and Regenerative Medicine Lab, Foundation Institute of Pediatric Research Città della Speranza, Padova (PD) - Veneto, Italy; Department of Women’s and Children’s Health, University of Padova, Padova (PD) - Veneto, Italy; Pediatric Gastroenterology, Digestive Endoscopy, Hepatology and Care of the Child with Liver Transplantation, Department of Women’s and Children’s Health, Padova University Hospital, Padova (PD) - Veneto, Italy; Department of Women’s and Children’s Health, University of Padova, Padova (PD) - Veneto, Italy; Stem Cells and Regenerative Medicine Lab, Foundation Institute of Pediatric Research Città della Speranza, Padova (PD) - Veneto, Italy; Department of Women’s and Children’s Health, University of Padova, Padova (PD) - Veneto, Italy; Pediatric Surgery Unit, Department of Women’s and Children’s Health, Padova University Hospital, Padova (PD) - Veneto, Italy; Department of Women’s and Children’s Health, University of Padova, Padova (PD) - Veneto, Italy; Stem Cells and Regenerative Medicine Lab, Foundation Institute of Pediatric Research Città della Speranza, Padova (PD) - Veneto, Italy

## Abstract

This review focuses on the crucial role of the intestinal epithelium in maintaining intestinal homeostasis and its significance in the pathogenesis of necrotizing enterocolitis (NEC) and inflammatory bowel diseases (IBD). NEC is a devastating neonatal disease, while IBD represents a global healthcare problem with increasing incidence. The breakdown of the intestinal barrier in neonates is considered pivotal in the development and progression of both disorders. This review provides an overview of the current state of in vitro, ex vivo, and animal models to study epithelial injury in NEC and IBD, addressing pertinent questions that engage clinicians and researchers alike. Despite significant advancements in early recognition and aggressive treatment, no single therapy has been conclusively proven effective in reducing the severity of these disorders. Although early interventions have improved clinical outcomes, NEC and IBD continue to impose substantial morbidity, mortality, and economic burdens on affected individuals and society. Consequently, exploring alternative therapeutic options capable of preventing and treating the sequelae of NEC and IBD has become a pressing necessity. In recent decades, extracellular vehicles (EVs) have emerged as a potential solution to modulate the pathogenic mechanism in these multifactorial and complex disorders. Despite the diverse array of proposed models, a comprehensive model to investigate and decelerate the progression of NEC and IBD remains to be established. To bridge the translational gap between preclinical studies and clinical applications, enhancements in the technical development of gut-on-a-chip models and EVs hold considerable promise.

## Graphical Abstract

**Graphical Abstract:**
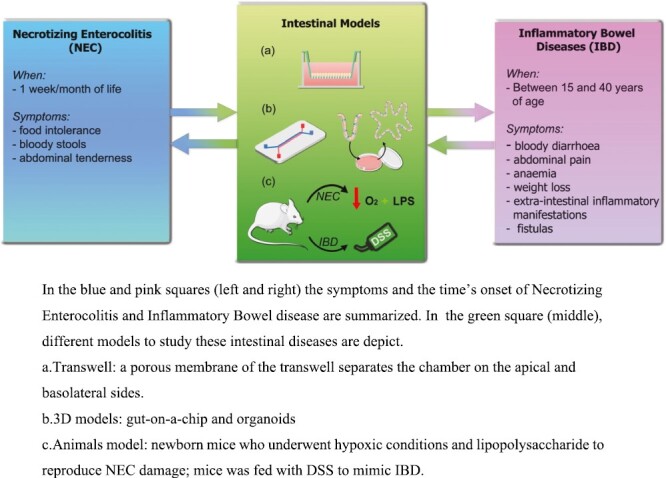

**Significance Statement**

The breakdown of the intestinal barrier brings together NEC and IBDs. Different models have been proposed to study the mechanisms of these disorders, underlying the importance of the inflammatory damage, gut permeability and infections in their development. Studying more in-depth intestinal permeability has led to increased knowledge of molecular mechanisms, helping scientists to discover some factors, including EVs, capable of modulating the damage in ex vivo models. Specifically, EVs have been recently demonstrated to be paramount in reducing damage across regenerative, anti-inflammatory, and antiapoptotic pathways in laboratory scenarios.

## Introduction

**Figure 1.**
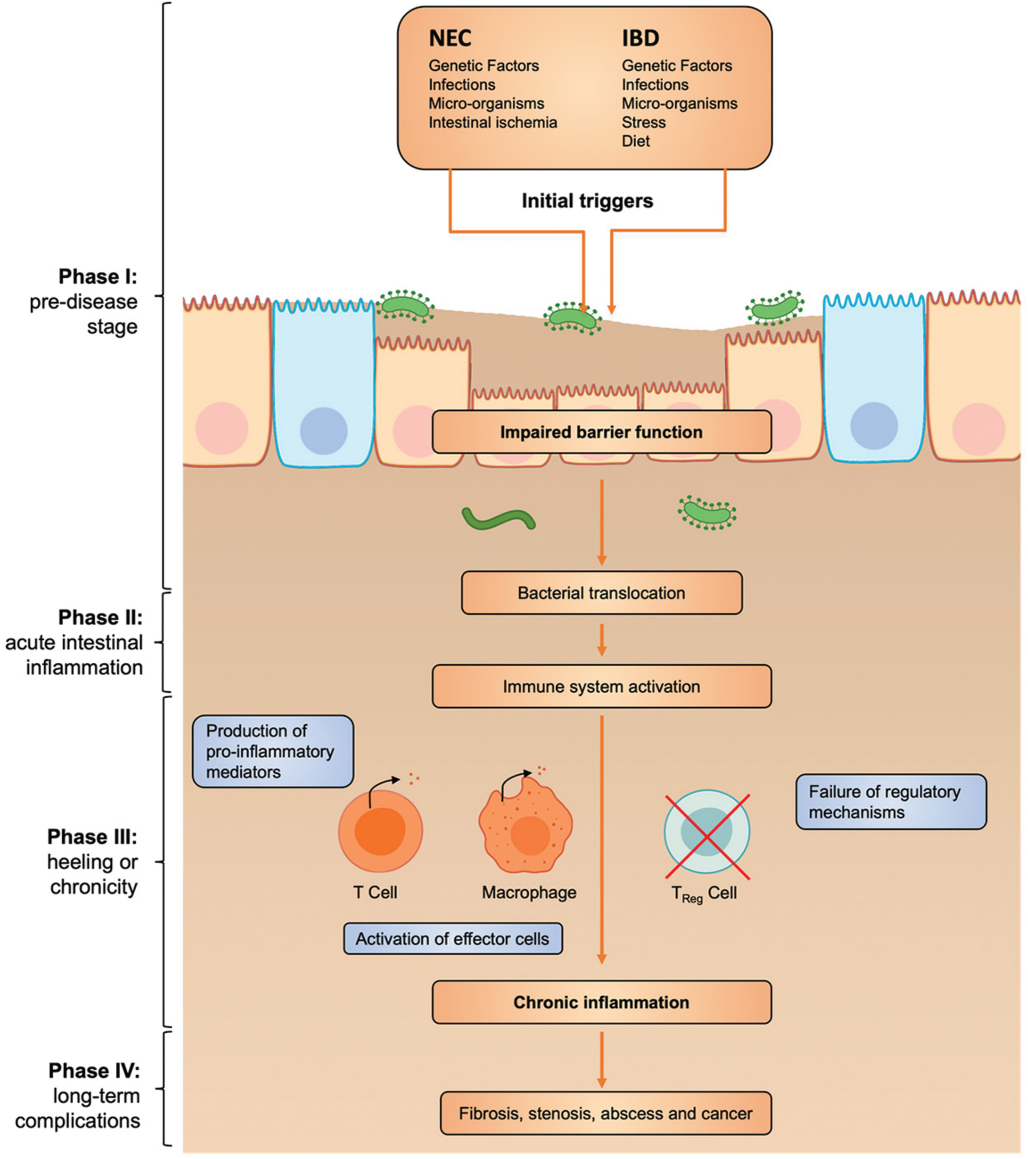
Schematic representation of the temporal phases of NEC and IBD pathogenesis, from the initial triggering to the chronic inflammation. Genetics and environmental factors are the initial triggers to impair the epithelial barrier. This impairment leads to bacteria translocation causing immune system activation. Failure of regulatory mechanisms and over-excess activation leads to chronic inflammation with complications (eg, fibrosis, stenosis).

The gastrointestinal (GI) tract is a tubular structure from the mouth to the anus and is responsible for the exchange of water and nutrients, occurring via a single layer. It encompasses several types of cells arranged in a series of fingers, like the lumen projections called villi and invaginations of mesenchyme called crypts.^1^ At the base of crypts, intestinal stem cells (ISCs) are crucial in intestinal integrity. The complex interaction among different types of cells with luminal microbiota is essential to maintain the critical balance between absorptive function and prevent bacteria translocation.^2^ When this balance is destroyed and the crosstalk between the neighborhood cells unpaired, different diseases might occur. The impairment in the intestinal barrier brings together necrotizing enterocolitis (NEC) and inflammatory bowel diseases (IBDs), which will be discussed in this review (Fig. 1) Breakdown of the intestinal barrier in neonates is thought to be a critical step in developing and progressing NEC, a devastating neonatal disease with a high mortality rate of approximately 30%-50% in very low-birth infants.^3,4^ Although many studies focused on NEC pathogenesis, it remains a major unsolved clinical challenge due to its extreme variability. The diseases’ inherent pathophysiologic complexity and etiological heterogeneity make creating models for primary research problematic. However, all experimental models showed an impairment in the intestinal barrier, which suggests that it may play a significant role in NEC pathogenesis.^5^ In young patients, an increase in intestinal permeability has been shown to precede IBD, characterized by chronically relapsing intestinal inflammation. These diseases, including Crohn’s disease (CD) and ulcerative colitis (UC), represent a global healthcare problem with continuous incidence.^6^ Based on the common clinicians’ and researchers’ questions, this review summarizes the current in vitro, ex vivo, and animal models to study epithelial injury in NEC and IBD. In addition, any current treatment available has been proven effective in reducing the severity of these disorders, even though early recognition and aggressive treatment have significantly improved the clinical outcome of both disorders. Because NEC and IBD still account for morbidity, mortality, and high costs for families and society, the exploration of alternative therapeutic approaches able to prevent the sequelae has become essential. In the last decades, extracellular vesicles (EVs), a group of lipid-embedded vesicles released by all cell types and with variable dimensions (50 to 10 000 nm), have emerged as a potential solution to modulate the damage in these multifactorial and complex disorders.^7^ Therefore, this review will also summarize their potential application in each model described.

## Models to Study Epithelial Damage

**Figure 2.**
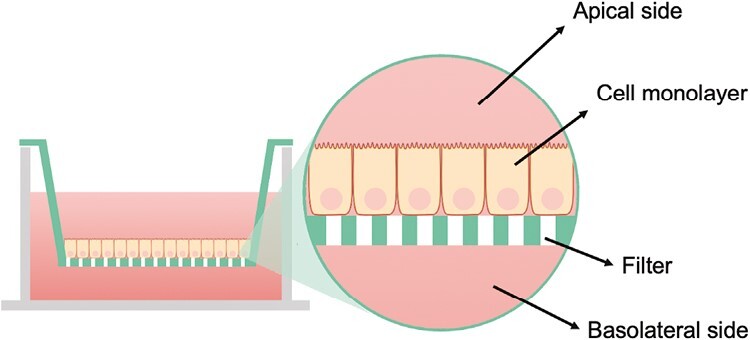
Transwell representation. Magnification of the Caco-2 forming the cell’s monolayer. Caco-2 polarized with the apical and basolateral sides typical of epithelial cells, helped by the transwell support.

The impairment of the intestinal barrier in NEC and IBD, characterized mainly by the disruption of tight junctions (TJ), contributes to their pathogenesis, promoting inflammatory response and bacterial translocation. Intestinal epithelial cells (IECs) are commonly used to study the epithelial barrier in NEC and IBD models. Human epithelial colorectal adenocarcinoma cells, Caco2 and HT29, have been widely used to investigate barrier dysfunction, defined by increased permeability and a reduction of transepithelial electrical resistance (TEER).^8^ Caco2 cells spontaneously differentiate in enterocytes after at least 21 days of culture, making a polarized layer with brush-border microvilli and expressing TJ proteins, such as Zonulin, Occludin, and Claudin. Conversely, HT29 differentiate only under specific nutritional and cultural condition/stimuli. Similarly, enterocyte disruptions, a hallmark of NEC pathology, are induced by treating IEC lines with lipopolysaccharide (LPS) or exposure to oxidative injury, producing hydrogen peroxide or inflammatory cytokines.^9^ In both models, proinflammatory cytokine levels significantly increase in the cell supernatant and decrease cell viability. Nevertheless, the monocultures do not show the complexity of the human intestine. Therefore, the coculture was proposed to generate a more reliable model closer to mimicking the complex geometry of intestinal architecture with different types of cells and scaffolds. To support cells toward apical and basolateral polarization in coculture models, a hydrophilic transwell device is used to enable the passage of water and solute (Fig. 2). The filters can come with different mesh sizes from 0.4 to 8 µm. To study permeability and transcellular transport, 0.4 µm filters are recommended.^10^ The transwell model can be a good alternative to study the damage effects of molecules against the barrier integrity; for instance, interferon-gamma (IFN-γ) and tumor necrosis factor alpha (TNF) were added into the transwell’s apical or basal side to simulate IBDs’ damage in vitro.^11^ Similarly, NEC is usually induced by treating IECs lines with LPS and H_2_0_2_ or inflammatory cytokines.^12^ Different readouts are available to assess the barrier integrity. TEER is a simple tool that gives real-time information about monolayer epithelial barrier integrity, composed of an electrode linked to the voltmeter.^13^ In physiologic situations, TEER values of the human colon are evaluated at about 50-400 ohm × cm^−2^, while Caco-2 values are higher, varying from 400 up to 1400-2400 ohm × cm^−2^.^13^ This high variability is due to the cell passage, temperature, medium composition, and electrode position.^13^ Alongside, immunofluorescence (IF) is a favorable readout to evaluate the integrity of TJ by evaluating biomarkers expressions, such as Zonulin, Occludin, and Claudin.^14-16^ Monoculture of intestinal-like cells has the advantage of targeting epithelial cells, while the lack of cell types and extracellular proteins make this model very limited.

## Models to Study the Influence of the Immune System and the Microenvironment in NEC and IBD

### In Vitro Models

Coculturing epithelial cells with other cell types allows the establishment of more complex models that mimic the in vivo conditions more effectively.^17^ Published data demonstrated that the adaptative immune system is required to induce intestinal inflammation and that defective immune regulation promotes chronic inflammation.^18-20^ Using cocultures is the simplest way to reproduce the interactions between the immune system and epithelial cells. One of the most exploited methods to mimic IBDs in vitro is coculture with epithelial cell-like and macrophage cell lines such as THP-1 and RAW. Specifically, THP-1 is a monocyte cell line isolated from the peripheral blood of an acute monocytic leukemia patient that differentiates in macrophages M1 after phorbol 12-myristate-13-acetate (PMA) stimulation.^18^ In inflammatory conditions, THP-1 secrete TNF and IFN-γ in the basal side, but due to the transwell mesh, these cytokines diffuse toward the apical side, stimulating the Caco-2 response.^19^ Cocultures help increase the system’s complexity and improve the physiologic behavior of a specific cell population.

### Ex Vivo Models

#### Organoids

##### Necrotizing Enterocolitis

The organoid-derived culture system provides a promising platform to study the ex vivo readouts of the epithelium in crosstalk in the NEC and IBD environment. Organoids can be maintained in culture for long time, mimicking the real-like organ.^20^ Different methods using intestinal crypts have been developed to allow the formation of villus-crypt-like structures capable of long-term self-renewal.^21^ One critical component is the leucine-rich repeat-containing G protein-coupled receptor 5 (LGR5+) stem cells. For their growth, these 3D structures needed Matrigel with a cocktail of enhancing agents essential for IECs expansion.^22^ Organoids have a spheroid shape surrounded by a cell layer and empty lumen nuclei. The epithelium structure is self-organized by crypts and villi, like the human intestinal epithelium. However, the apical surface of the epithelium is enclosed within the spheroid differently from human enterocytes, where the external milieu is in contact with the enterocyte apical side.^23^ To overcome this challenge, some studies used microinjection techniques to introduce external agents into the organoid lumens even through mucus accumulation and cell debris, preventing good accessibility of the epithelial surface. Co et al developed a method to reverse the epithelial polarity of the organoids producing apical-out organoids, enabling the addition of external agents into culture media to simulate the in vivo damage.^23^ Sequentially, intestinal organoids from humans were established.^21^ This ex vivo model has proven to be a robust tool for studying the complex physiopathology of NEC and testing possible therapeutic targets. Sodhi et al showed the role of intestinal epithelial toll-like receptor 4 (TLR4) in NEC development using organoids from TLR4-deficient adult mice.^24^ As neonatal epithelium differs from adults, recent studies performed with neonatal mice samples showed that exposing organoids to hypoxia and LPS are both needed as stress factors to induce intestinal injury in vitro, confirmed by an increase of Interleukin-1, Interlukin-6, TNF and by a downregulation of tight-junction proteins.^25^ Enteroids (derived from the small intestine) or colonoids (derived from the colon) are 3D miniaturized structures that closely resemble the structure and function of the native intestinal epithelium. By culturing enteroids in the lab, researchers can study and manipulate the behavior of intestinal stem cells, investigate disease mechanisms, and explore personalized medicine approaches for gastrointestinal disorders.^26-28^ Werts et al developed a NEC model by exposing enteroids to NEC bacteria or hypoxia. However, a reduction in proliferation was observed in NED-induced enteroids, consistent with natural epithelial differentiation in the intestine. In addition, they proposed a NEC-in-a-dish model by subjecting enteroids to hypoxia and NEC bacteria, mimicking NEC in an ex vivo setting. In this model, differentiated intestinal epithelium showed disrupted tissue architecture, increased inflammatory cytokines, and enhanced necroptosis activation. Notably, inhibiting necroptosis with Nec1s partially prevented these damaging effects, inflammatory cytokine induction, and necroptosis activation.^29^ Recently, new techniques have been proposed to create human intestinal-derived organoids (HiOs), not only derived by normal ileal biopsies but also from infants affected by NEC who underwent surgery for severe NEC.^30^ The HiOs have been used to simulate NEC damage in ex vivo culture and their availability to test potential therapeutic agents.^25^ The organoids derived by infants affected by NEC were used to prove an impaired regeneration capability and a higher propensity to differentiate compared to ex vivo damage.^31^

##### Inflammatory Bowel Disease

In 2009, Sato et al described the first intestinal organoid from mice proving that these crypt-villus structures, supported by Matrigel with a high complex medium culture, were able to differentiate into all intestinal cell types, summarizing the organization and function of the small bowel in vivo.^32^ On the other hand, organoid-based models of IBD have been established from the inflamed-mucosa-derived patients showing epithelial alteration with decreased expression of tight-junction proteins, slow-growth, altered polarization, and upregulation of proinflammatory cytokines, such as monocyte Chemoattractant Protein-1 (MCP-1) and TNF.^33^ In addition, organoid cultures from healthy subjects can explore the specific biological role IBD-associated proinflammatory factors play in epithelial barrier integrity. Coculturing organoids with immune cells help investigate specific interaction between intestinal organoids mimicking IBD damage and immune response. For instance, Ihara et al has proposed a model coculturing intestinal organoids with lamina propria leukocytes and bone marrow-derived dendritic cells from CD11c-cre Tgfbr2^fl/fl^ mice, leading to dysbiosis and intestinal inflammation.^34^ The IBD organoids models have recently been used to identify possible therapeutic targets. Deuring et al proposed rifampicin or other pregnane X receptor ligands as a novel treatment in IBD patients, especially when the nuclear factor kappa beta (NF-κB) pathway is hyperactivated.^35^ Therefore, HiOs from specific patients can test different therapeutic targets to personalize treatment in these diseases, reducing adverse effects. In addition, the long-term expansion capability of organoids is opening various possibilities to create disease-derived organoid biobanks. This scenario might represent a valuable resource for clinical applications such as omics and drug analysis in precision medicine.

#### In Vivo Models

##### Necrotizing Enterocolitis

Mice and rats are the most common in vivo models used in NEC and IBD so far. The first animal model used to mimic NEC was described by Barlow et al in 1974, in which mice were fed with hyperosmolar feeding and subjected to intermittent hypoxic conditions.^36^ Caplan et al developed a rat model to study intestinal injury in pups caused by formula feeding and hypoxia.^37^ They emphasized that hypoxia played a crucial role in inducing these stress-related conditions.^37^ However, this model has 2 main limitations, despite its widespread use in studying experimental NEC. The first one is that the pups do not survive beyond a few days,^20^ and the second one is related to the fact that the most common reagents targets are made for murine models. Recently, researchers successfully induced NEC in mouse by utilizing formula feeding and subjecting the mice to a cold hypoxic environment. This mouse model can be genetically modified, making it a valuable tool for studying the involvement of different genes in developing NEC.^20,38^ The pioneering work in creating a transgenic NEC model was carried out by Jilling et al^39^ Their study utilized a transgenic strain of mice called C3H/HeJ (TLR4 transgenic). They identified Gram-negative bacteria’s crucial role in NEC’s pathogenesis, although TLR4-mutant mice did not develop NEC.^39^ This finding shed light on the specific role of TLR4 and its connection to the disease’s development. Thus, while neonatal mouse models of NEC have been extensively utilized, they present challenges such as a steep learning curve and a notable rate of technical mortality.^40^ Consequently, researchers have explored alternative options, turning to larger animal’ models, like pigs. Piglets have a gastrointestinal tract that closely resembles the human intestine, making them attractive candidates for studying the effects of probiotics and breast milk in mitigating the severity of NEC damage.^41^ However, the high comes with its limitations, including high costs, substantial space requirements for housing, and the inability to be genetically modified, which restricts its suitability for specific research studies.^31,42^ The FITC-dextran method evaluates intestinal barrier function and permeability. Utilizing a fluorescently labelled compound, FITC-dextran, which is safe and water-soluble, to assess the integrity of the intestinal barrier. However, any compromise in barrier integrity results in the leakage of FITC-dextran into the systemic circulation. The level of fluorescence in collected blood samples serves as a quantifiable indicator of gut permeability, thereby providing valuable insights into the health status of the intestinal mucosa.^43,44^ Indeed, investigations centered on bacteria translocation studies within in vivo models, focusing on GI inflammation and epithelial damage, have significantly contributed to elucidating the phenomenon of bacteria migrating from the gut to other organs.^45,46^ By examining these processes, valuable insights have been gained into the consequences of a compromised gut barrier on various diseases, particularly those associated with gut inflammation. Consequently, such studies identify potential therapeutic targets for conditions linked to gut inflammation.

##### Inflammatory Bowel Disease

One of the most used animal models to study IBD is the chemically induced IBD mouse model, which involves dextran sulfate sodium (DSS) damage. This model has been shown to induce tight-junction disruption, leading to downregulating ZO-1 and Occludin proteins and increased epithelial permeability.^47^ Consequently, it mimics some key pathological features observed in human IBD. Moreover, this mouse model offers the advantage of exploring the influence of immunity on the resolution of inflammation and the regeneration mechanism. Using flow cytometry, researchers can analyze the polarization of T cells and macrophages population, specifically toward Th1/Treg or M1/M2 phenotypes. In addition, the inflammation and/or regeneration levels can be evaluated through various techniques, including immunofluorescence, histological analysis, and clinical assessments.^48^ Indeed, some researchers opt for genetically modified IBD models to investigate specific intracellular pathways implicated in IBD pathogenesis. For instance, in interleukin-10 (IL-10), knockout mice developed spontaneous colitis, displaying disruptions in tight junction, and increased permeability.^49^ This emphasized the protective role of IL-10 in IBD pathogenesis. The genetically modified IBD models are important to study the influence of the microbiota on colitis development, mucosal homeostasis, and the relevance of the immune system. Studies focused on modulating the damage have indicated that administering a broad spectrum of antibiotics can lead to decreased intestinal inflammation and reduced concentrations of selective species of bacteria.^50^ As NEC and IBD are complex diseases with multiple contributing factors, animal models offer valuable insights into monitoring the morphological changes in tissues and genetic pathways. However, animal models have certain limitations, as they cannot fully replicate the diverse conditions found in the human body, including variations in microbiota and chronic damage. Considering ethical concerns and clinical implications associated with the use of animal models, the search for viable alternatives becomes essential. Ex vivo models, such as organoids and gut-on-chip systems, have emerged as attractive options. These models offer the potential to mimic human physiology and interactions better, providing a valuable approach to studying the complexities of NEC and IBDs and potentially serving as effective tools for exploring therapeutic strategies.

## Models to Study the Influence of Microbiome in NEC and IBD

The interaction between the host and the gut microbiota is a crucial factor in the NEC and IBD’s pathogenesis. To study effectively, intestinal organoids have emerged as a valuable tool due to their ability to mimic the complex intestinal microenvironment. However, it is important to acknowledge the risk of culture contamination when working with organoids. To address this challenge, posed by culturing anaerobic microbiota and the intestinal epithelium simultaneously, research has turned to compartmentalized platforms. These platforms utilized semipermeable membrane to minimize oxygen diffusion, creating separate environments catering to the needs of both the anaerobic microbiota and the intestinal epithelium. By employing such compartmentalized systems, researchers have achieved superior conditions for studying host-bacterial communication and gaining deeper insights into the pathogenesis of these intestinal disorders.^51^ However, these compartmentalized platforms used for studying host-microbial interactions in intestinal organoids have been noted to exhibit a limitation, namely the absence of direct contact of microbes with epithelial and immune cells. This direct contact is deemed crucial as it plays a significant role in influencing the functionality of intestinal epithelium and vice-versa.^52^ To overcome this limitation, Puschhof et al proposed a potential solution by suggesting the microinjection of microbes into the organoid lumen, thus replicating the natural habitat of the microbiota.^53^ However, it is essential to consider the practical implications of such microinjection’s techniques. Handling these organoids through microinjection is considered time-consuming and may impose certain restrictions on studies that involve subsequent genetic manipulation of the organoids after their exposure to specific pathogens. Numerous *gut-on-chip* models have been micro-engineered to create an in vitro environment that allows for the coexistence and interaction of the human intestinal epithelium, immune cells, capillary endothelium, and microbes. These models are designed to replicate peristalsis-like motions simulating the physiological conditions within the human gut.^54^ Lanik et al proposed a novel *on-a-chip* model specially tailored to explore the intricate pathogenetic mechanisms underlying the development of NEC. The aim was to gain a deeper understanding of NEC and eventually develop precision medicine approaches by generating patient-specific treatments.^55^ Similarly, *gut-on-chip* technology holds promise as a reliable platform that may potentially replace the conventional DSS-induced colitis model in mice. This advancement aims to bridge the gap between animal studies and clinical research in the field of IBD research, offering more relevant insights for therapeutic interventions.^56^ Kim et al took an already-described human gut-on-a-chip model and adapted it to develop a disease model of intestinal bacterial overgrowth and inflammation. Through this model, they investigated the individual and combined contributions of various factors, including commensal and pathogenic microbes, LPS, immune cells, inflammatory cytokines, vascular endothelial cells, and mechanical forces, to the impairment of the epithelial barrier and the development of intestinal inflammation.^57^ Furthermore, they explored the potential of combinations of antibiotics in suppressing intestinal injury and promoting the recovery of the intestinal barrier. However, it is worth noting that the antibiotics used in this study differed from those commonly used in clinical practice. Thus, future experiments must examine the effects of commonly used antibiotics to advance personalized medicine approaches.^58^ These platforms address several limitations seen in vitro and animal models, such as discrepancies between human and mouse physiology and the variability of microbial components. By using patient-specific epithelium, these models can be promising first step toward developing personalized medicine strategies for gastrointestinal diseases. However, it is crucial to acknowledge that these approaches are highly complex and labor-intensive, which can lead to challenges in reproducibility (Tables 1 and 2). Despite their potential, further refinements and standardization may be necessary to enhance the practical applicability of these models in research and potential clinical use.

**Table 1.** Summary of disease models and EVs actions.

NEC and IBD models	Pro	Cons	EVs applications	References
In vitro -Cell culture -Transwell model	• Real-time monitoring • Direct targeting of epithelial cells • Low costs	• Lack of environmental complexity • Lack of communication with other cells (classic 2D) • Long differentiation timing relative to epithelial cells	• Regulation of tight junction expression protein • Modulation of TLR expression	• ^8,9,14-16^
Ex vivo -Organoids -Gut-on-chip	• rRobust tool for disease modelling • Long-term culturing gene manipulation possibilities • Availability to use patients’ tissue	• Lack of other vital elements of the intestine (vascular, lymphatic, immune system) for organoid models • Difficult to reproduce model for gut-on-chip	• Mitigation of epithelial damage • Reduction of epithelial apoptosis	• ^20-22,31,54-57,58^
In vivo -Animal models	• Complex system • Very similar to the physio-pathological situation in humans • Availability of several strains (mouse)	• Ethical concerns • Bias of species • Clinical considerations • Difficulty in chronicity reproduction	• Enhancement of cellular regeneration • Modulation of inflammatory damage	• ^38,40,48-50^

**Table 2.** Summary of underlying pathogenetic mechanisms and possible models to study NEC and IBD.

Topics	Target	Model applications
-Barrier function impairment	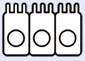 Epithelial cells	-Monoculture, transwell model, animal models, organoids, gut-on-chip
-Loss of immunoregulatory response -Inflammation	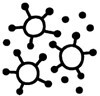 Immune system	-Animal models, gut-on-chip
-Dysbiosis, specific microbial triggers	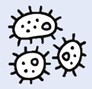 Microbiome	-Organoids, gut-on-chip
-Understanding variability in a clinical course	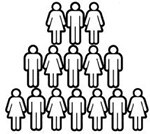 Personalized medicine	-Gut-on-chip

## EVs as a Medical Approach for NEC and IBD Treatment

Recently, a novel player has emerged in studying IBD and NEC, the EVs. EVs have been studied as a therapeutic approach to recovering damaged tissue. These tiny vesicles secreted by cells are involved in intercellular communication and play diverse roles in modulating cellular functions and tissue homeostasis. EVs derived from various cell sources, such as mesenchymal stromal cells (MSCs) and commensal bacteria, have shown promising effects in reducing inflammation, promoting tissue repair, and enhancing barrier integrity.^7,15,16^ Integrating EVs into both ex vivo and in vivo models offers a powerful approach to studying the complex pathogenesis of IBD and NEC. In ex vivo organoid models, EVs have emerged as a promising field and have garnered increasing attention in recent years. In various ex vivo and in vivo models, EVs have demonstrated anti-inflammatory and proregenerative effects, as evidenced by flow cytometry analysis of macrophage and lymphocyte, histological assessment, and evaluation of cytokines ratio.^48^ Specifically, EVs derived from human mesenchymal stromal cells (MSC-EVs) and commensal bacteria (CB-EVs) have been investigated for their potential to improve barrier integrity by regulating TJ genes and proteins, thus preventing intestinal barrier breakdown.^14,15,48,59^ CB-EVs exert positive effects by interacting with pattern recognition receptors (PRRs) and modulating the expression of toll-like receptors (TLRs), which play an essential role in regulating inflammation and intestinal homeostasis.^16^ Furthermore, MSC-EVs have been reported as paramount mediators in tissue homeostasis and restoration after injury.^31^ Numerous studies have provided evidence of the effectiveness of EV injection in reducing mucosal damage, as shown by increased cellular proliferation, decreased intestinal inflammation, and enhanced cellular regeneration.^31,42^ Similarly, in ex vivo IBD models, EVs have been utilized to mitigate epithelial damage and decrease epithelial apoptosis.^36^ The discoveries made through these studies indicate that EVs hold great promise as a potential therapeutic option for mitigating intestinal damage. As the research on EVs in NEC and IBD continues to expand, it opens exciting possibilities for developing innovative treatment approaches tailored to individual patients. These debilitating gastrointestinal disorders could see new hope through the harnessing of EVs’ therapeutic potential.

## Conclusions and Future Perspectives

In conclusion, the impairment of the intestinal barrier is a critical mechanism underlying NEC and IBD pathogenesis. This breakdown disrupts not only intestinal homeostasis but also has systemic effects on the patient’s overall health. Consequently, various in vitro, ex vivo, and in vivo models have been proposed to study the intricate mechanisms involved in these disorders, focusing on inflammatory damage, gut permeability, and infections as key in their development. The study of intestinal permeability has provided valuable insights into molecular mechanisms, leading to the discovery of factors, such as EVs, capable of modulating damage in ex vivo models. Recently, EVs have shown significant potential in reducing damage through regenerative, anti-inflammation, and antiapoptotic pathways in laboratory settings. However, the quest for a comprehensive model to study these disorders and effectively slow disease progression continues. Advancements in gut-on-a-chip models, including incorporating immune, vascular, and nervous systems, along with the microbiome, could represent a promising new frontier in understanding these devasting diseases. This multifaceted approach may uncover successful strategies for preventing and treating NEC and IBD. In addition, engineered EVs containing “protective” molecules hold promise as potential therapeutic interventions and should be thoroughly validated in laboratory settings. By harnessing these cutting-edge technologies and knowledge, researchers aim to advance the understanding and management of NEC and IBD, offering hope for improved patient outcomes.

## Data Availability

The data underlying this article will be shared on reasonable request to the corresponding author.
